# Addendum: Transcriptomics and proteomics reveal two waves of translational repression during the maturation of malaria parasite sporozoites

**DOI:** 10.1038/s41467-021-27767-7

**Published:** 2022-01-06

**Authors:** Scott E. Lindner, Kristian E. Swearingen, Melanie J. Shears, Aswathy Sebastian, Michael P. Walker, Erin N. Vrana, Kevin J. Hart, Allen M. Minns, Istvan Albert, Photini Sinnis, Robert L. Moritz, Stefan H. I. Kappe

**Affiliations:** 1 Department of Biochemistry and Molecular Biology, The Huck Center for Malaria Research, Pennsylvania State University, W225 Millennium Science Complex, University Park, PA 16802 USA; 2 Institute for Systems Biology, 401 Terry Avenue N, Seattle, WA 98109 USA; 3 Department of Molecular Microbiology & Immunology, Johns Hopkins Bloomberg School of Public Health, 615 N. Wolfe Street, Baltimore, MD 21205 USA; 4 Huck Institutes of the Life Sciences, Pennsylvania State University, University Park, PA 16802 USA; 5 Center for Global Infectious Disease Research, Seattle Children’s Research Institute, 307 Westlake Avenue N. Suite 500, Seattle, WA 98109 USA; 6 Department of Global Health, University of Washington, Seattle, WA USA; 7 Present Address: Department of Laboratory Medicine, University of Washington, 1959 NE Pacfici St., Seattle, WA 98195 USA

**Keywords:** Parasite biology, Proteomics, Transcriptomics

Addendum to: *Nature Communications* 10.1038/s41467-019-12936-6, published online 31 October 2019.

In the original version of the Article, an inadvertent operator error resulted in our processing of the RNA-seq data to not account for the correct strandedness of the mapped reads (e.g., a single toggle was not changed from the default). Here with the key contributions of our bioinformatician colleagues (now added as co-authors), we have thus reanalyzed these RNA-seq data (described below) and we provide the downstream interpretations based upon them in this Addendum. Importantly, upon a comparison of the rank abundance of the sense and antisense mapped reads, we observed strong correlations for both *P. falciparum* and *P. yoelii* in both stages of sporozoite development (Supplementary Fig. 1). Perhaps due to this, the major conclusions of the original Article still are valid and do not require modification. However, the new analyses required modification of the composition of some gene lists. In addition, we have now also identified and accounted for run-in transcription through the application of a Transcript Integrity Number (TIN) metric that is applied to all transcripts to make these datasets more robust^1^. We provide those corrections to the gene lists and relevant figures and Supplementary Files here.

First, using *P. yoelii* 17XNL strain parasites we have now identified 2795 and 2402 RNAs with detectable and unambiguous sequence reads that pass all inclusion thresholds (including minimum read count >20) that are present in oocyst sporozoites and salivary gland sporozoites, respectively (versus 4195 and 3887 RNAs previously identified) (Supplementary Data 1). Similarly, with *P. falciparum* NF54 strain parasites, we identified 2408 and 2166 RNAs in oocyst sporozoite and salivary gland sporozoite stages, respectively (versus 3535 and 3575 RNAs previously identified). The most abundant mRNAs in these stages remain largely as previously reported, and the same extreme swings in mRNA abundance across oocyst sporozoite and salivary gland sporozoite stages are evident for some transcripts (e.g., *py17x_0514800*, >90-fold; *py17x_1354300*, 297-fold) including those of the upregulated in infectious sporozoites (UIS) family (e.g., *pyuis4*, ~1900-fold increase in abundance) (Fig. 1, Supplementary Data 2, and Supplementary Data 3). As noted above, we also observed substantial antisense transcription in *Plasmodium* sporozoites. Even after stringent filtering was applied to account for run-in transcription that occurs when a neighboring gene is transcribed into the annotated gene feature, we detected 306 and 327 antisense transcripts in *P. falciparum* oocyst sporozoites and salivary gland sporozoites respectively, as well as 479 and 410 antisense transcripts in *P. yoelii* oocyst sporozoites and salivary gland sporozoites, respectively. A comparison of the ranked abundance of antisense transcripts and the ranked abundance of sense transcripts revealed a strong statistically significant correlation (Supplementary Fig. 1). Moreover, some of these antisense transcripts are differentially expressed across sporozoite stages and across species, which certainly warrants further study to understand if and how these affect biologically important processes (Supplementary Fig. 2 and Supplementary Data 3).

**Fig. 1:**
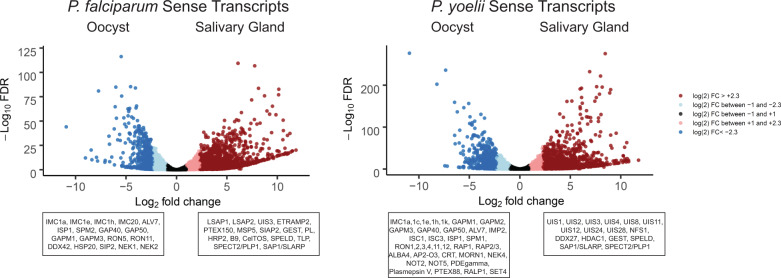
Comparative sense transcriptomics of oocyst and salivary gland sporozoites. RNA from purified *P. falciparum* or *P. yoelii* sporozoite isolated from oocysts or the salivary glands was assessed by RNA-seq, and transcript abundances compared by DEseq2. Sense transcripts are plotted based upon fold change (between oocyst sporozoites and salivary gland sporozoites) and false discovery rate (FDR), with mRNAs shaded in lighter (+/− 1 to 2.3 log_2_ fold change) or darker shakes (>2.3 log_2_ fold change). Notable, differentially expressed transcripts are labeled in boxed under the plot.

Using these data to assign upregulated in Oocyst Sporozoites (UOS) and UIS mRNAs resulted in a more stringent list of 104 UOS mRNAs and 80 UIS mRNAs in *P. yoelii*, and 61 UOS mRNAs and 63 UIS mRNAs in *P. falciparum* (Supplementary Data 3). As before, the most well-studied genes remained classified the same way in this reassessed data, including a core set of the most abundant *P. yoelii* UIS transcripts: *uis1*, *uis2*, *uis3*, *uis4*, *uis7*, *uis8*, *uis12, uis24*, and *uis28*. In addition, we observed an additional 88 *P. yoelii* and 47 *P. falciparum* transcripts in the seventh to the ninth decile that increase >10-fold in abundance in salivary gland sporozoites vs. oocyst sporozoites. These include *pflsap1*, *pfmsp5*, *pfsera9*, *pfddx60*, an ApiAP2 (*pf3d7_1342900*), *pyuis20*, *pycdc6*, *pyhdac1*, *pydbp4*, *pydbp10*, *pynek1*, *pysir2a*, and *pypsop24* (Supplementary Data 4). As no changes to the analyses of our proteomic data were required, we were able to compare the original proteomic datasets with these revised transcriptomic datasets to assign gene products as UOS mRNAs, UIS mRNAs, UOS proteins, and/or UIS proteins (Fig. 2). As reported in the original Article, few genes receive the same UOS designations across species (e.g., 25 UOS mRNAs, 6 UOS proteins), and few that are both UOS mRNAs and proteins (2 in *P. falciparum*, 15 in *P. yoelii*). While there are no syntenic genes that have a UOS mRNA and UOS protein designation across species, the well-characterized TREP/UOS3 nearly meets this level except that it is in the 88th percentile for RNA abundance in *P. falciparum* (Supplementary Data 1). Also as found in the original Article, few gene products receive the same UIS designations across species (e.g., 15 UIS mRNAs and 21 UIS proteins). However, those that do are well-characterized gene products that are important to the activities and required functions of salivary gland sporozoites (e.g., p36, SPECT2/PLP1, GEST, CelTOS, and SPELD).

**Fig. 2:**
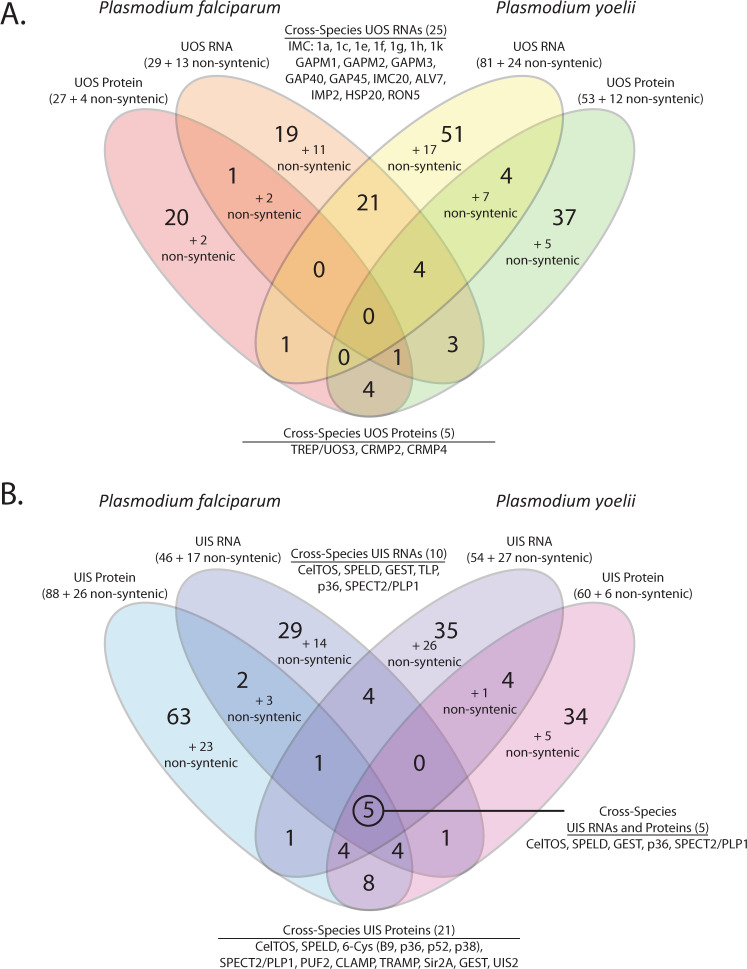
Comparisons of UOS and UIS Gene Products. Gene products in the top decile of abundance that were 5-fold (RNA) or 6-fold (protein) more abundant in either oocyst sporozoites or salivary gland sporozoites were denoted as UOS or UIS gene products, respectively. Comparisons across molecule types and species for (**A**) UOS and (**B**) UIS identify many gene products that are critical/essential to sporozoite development and/or transmission. Gene products that are similarly regulated across species, transcriptional levels, and/or translational levels are indicated.

Finally, through comparisons of our reanalyzed transcriptomic and original proteomic datasets using the same metrics as in the original article, we confirmed that *Plasmodium* sporozoites extensively use translational repression, and undergo two programs of translational repression across their development (Supplementary Data 5 and 6 and Supplementary Figs. 3 and 4). For simplicity, we have abbreviated these as Program 1 (“TR-oospz to UIS Proteins Program”) and Program 2 (“Pan-Sporozoite TR Program”). Well-characterized Program 1 transcripts in both species include SPECT2/PLP1, CelTOS, SPATR, CLAMP, GEST) (Supplementary Data 7). Notable Program 2 transcripts for both species include ApiAP2s, PAIP1, CAF1, NOT4, CDPK6, CHD1, FIKK8, LSD2, MTRAP, SAP1/SLARP, and STK2 (Supplementary Data 8). In addition, in *P. yoelii*, several of the historically defined UIS mRNAs (UIS4, UIS8, UIS12, UIS28), several ApiAP2s, TLP, ISWI, GRASP, ZIPCO, DBP9, DDX23, DDX27, GEXP15, and others are regulated by Program 2. In *P. falciparum*, several ApiAP2s (including AP2-I and AP2-O), LSAP2, MSP4, SET9, ETRAMP5, ETRAMP10.3, EXP1, PV1, PTEX150, MAPK1, and other regulator proteins are also controlled by Program 2.

Together, the reanalyzed data continue to support the major finding of the original article: *Plasmodium* sporozoites extensively use two orthogonal programs of translational repression. Moreover, these data also support our model that Program 1 prepares *Plasmodium* sporozoites for the transmission event and traversal through the host skin and vasculature, whereas Program 2 is used in preparation for parasite development in the host liver.

## Revised RNA-seq analyses

In conducting these revised analyses, the Illumina RNA-seq data was mapped to the respective reference genomes (*P. yoelii* 17XNL strain, plasmodb.org v30; *P. falciparum* 3D7 strain, plasmodb.org v30) using hisat2 (version 2.1.0)^2^ specifying --rna-strandness R and --max-intronlen 5000 parameters. Coverage files were generated and the mapped data was visualized and inspected in Integrative Genomics Viewer (IGV)^3^. Oocyst sporozoite samples had a lower mapping rate (average mapping rate: *P. falciparum* oocyst sporozoites—6.76%, *P. yoelii* oocyst sporozoites—6.23%) compared to salivary gland sporozoite samples (average mapping rate: *P. falciparum* salivary gland sporozoites—32.49%, *P. yoelii* salivary gland sporozoites—17.03%). Reads mapped to genes (annotations from PlasmoDB.org v30 for both *Plasmodium yoelii* 17XNL strain and *Plasmodium falciparum* 3D7 strain) were counted using htseq-count (version 0.12.4)^4^ specifying --stranded=reverse and --minaqual = 30 parameters. DESeq2 (version 1.26.0)^4^ was used to normalize the raw read counts and to obtain the differentially expressed genes between the conditions. Transcript Integrity Number (TIN)^1^ for each gene was calculated to further filter out the differentially expressed genes that do not have coverage evenness across the entire length of the gene. To calculate TIN, transcriptome was built from the annotation files and pseudobam files were generated using Kallisto (version 0.46.1, parameters used: specifying --rf-stranded --single -l 150 -s 20 -b 10)^4^. Read depth at each position in the transcript was obtained using samtools depth command and TIN was calculated as described (custom python scripts are provided as Supplementary Code 1)^4^. For genes with multiple transcripts, the maximum of the transcript TINs was considered as the integrity value for the gene. Differentially expressed genes were selected specifying FDR <0.05, read count >20, and where at least one of the two samples had a TIN >40. Antisense differential expression results were obtained by repeating the procedure but specifying antisense strand when required ie, --stranded =yes in htseq-count and --fr-stranded in kallisto. All commands that were used in this analysis are included in a Makefile provided in Supplementary Code 1.

## Supplementary information

Supplementary Code 1

Supplementary Data 1

Supplementary Data 2

Supplementary Data 3

Supplementary Data 4

Supplementary Data 5

Supplementary Data 6

Supplementary Data 7

Supplementary Data 8

Supplementary Figures S1-S4
