# Supplementary material for: Transcriptomics and proteomics reveal two waves of translational repression during the maturation of malaria parasite sporozoites
Source: Nat Commun. 2019 Oct 31;10:4964. doi: 10.1038/s41467-019-12936-6 (PMC6823429; doi:10.1038/s41467-019-12936-6)
Supplement: Supplementary file 1 — Supplementary Information [file 41467_2019_12936_MOESM1_ESM.pdf]

Transcriptomics and proteomics reveal two waves of translational repression during the maturation of malaria parasite sporozoites

Lindner and Swearingen *et al.*

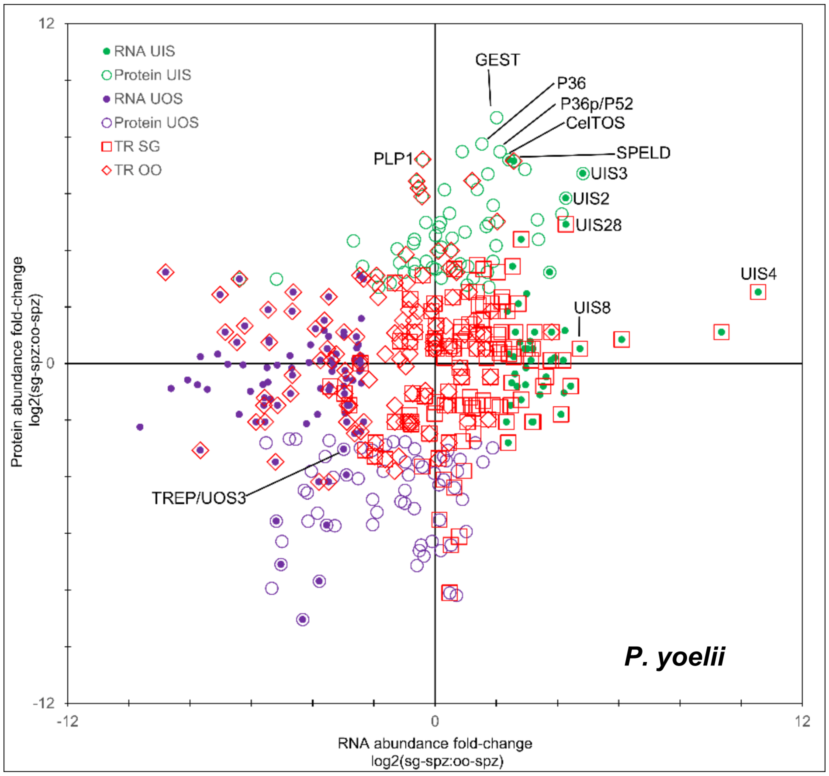

RNA UIS
Protein UIS
RNA UOS
Protein UOS
TR SG
TR OO
GEST
P36
P36p/P52
CelTOS
SPELD
UIS3
UIS2
UIS28
PLP1
UIS4
UIS8
TREP/UOS3
P. yoelii
Protein abundance fold-change
log2(sg-spz:oo-spz)
RNA abundance fold-change
log2(sg-spz:oo-spz)
12
0
-12
-12
0
12

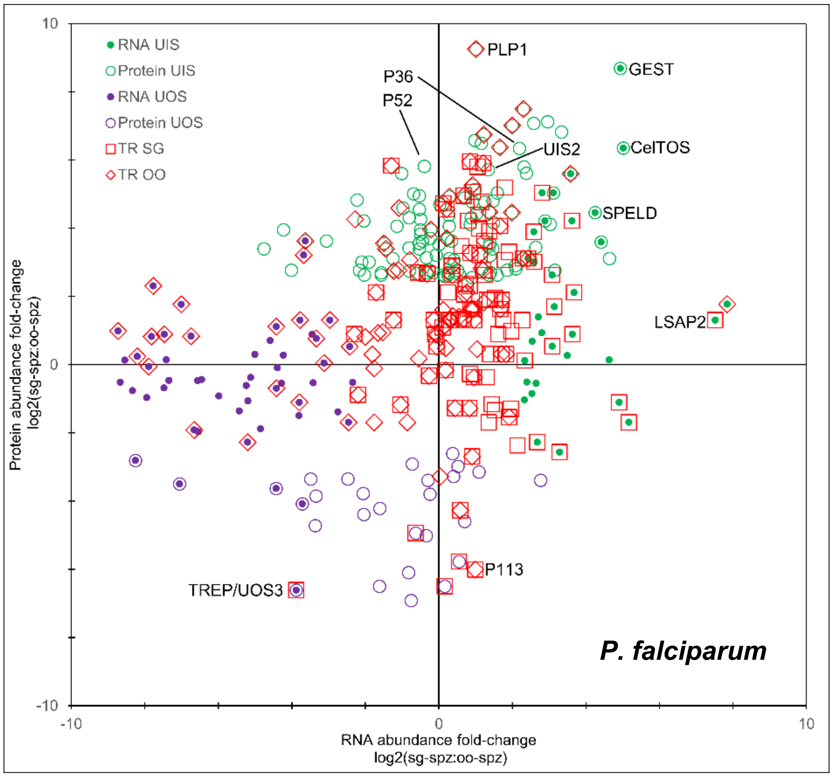

RNA UIS
Protein UIS
RNA UOS
Protein UOS
TR SG
TR OO
PLP1
GEST
P36
P52
UIS2
CelTOS
SPELD
LSAP2
P113
TREP/UOS3
P. falciparum
Protein abundance fold-change
log2(sg-spz:oo-spz)
RNA abundance fold-change
log2(sg-spz:oo-spz)
10
0
-10
-10
0
10

Supplementary Figure 1: Relative abundance change in protein with respect to relative abundance change in mRNA. Shown are genes that were categorized as at least one of the following: mRNA was upregulated in infectious sporozoites (UIS, filled green circles), protein was UIS (open green circles), mRNA was upregulated in oocyst sporozoites (UOS, closed purple circles), protein was UOS (open purple circles), transcript was translationally repressed in oocyst sporozoites (open red diamonds), or transcript was translationally repressed in salivary gland sporozoites (open red squares). Additionally, data was only plotted if fold-change ratios were obtained for both mRNA and protein. Multiple translational repression programs can be observed. For example, the invasion-related proteins CelTOS (Cell traversal for ookinetes and sporozoites) and SPELD (sporozoite protein essential for liver stage development) are UIS at both the mRNA and protein levels in both *P. yoelii* and *P. falciparum*. GEST (gamete egress and sporozoite traversal protein) is similarly regulated, though the increase in RNA is more mutated in *P. yoelii*. The invasion-related proteins P36 and P52 are also among the proteins that exhibit no change in mRNA between oocyst and salivary gland stages, but are UIS at the protein level presumably in preparation for their roles in hepatocyte invasion and development. PLP1 (perforin-like protein 1), which is required for cell traversal prior to invasion, is translationally repressed in oocyst sporozoites, but de-repressed in salivary gland sporozoites, thereby becoming a UIS protein under the TR-oo to UIS program we describe here. Proteins under the Pan-Sporozoite repression program include LSAP2 (liver stage-associated protein 2) in *P. falciparum* and UIS4 in *P. yoelii*. Both proteins are important for development of liver stages, and both proteins are encoded by mRNAs that are UIS but remain translationally repressed, presumably in preparation for de-repression.

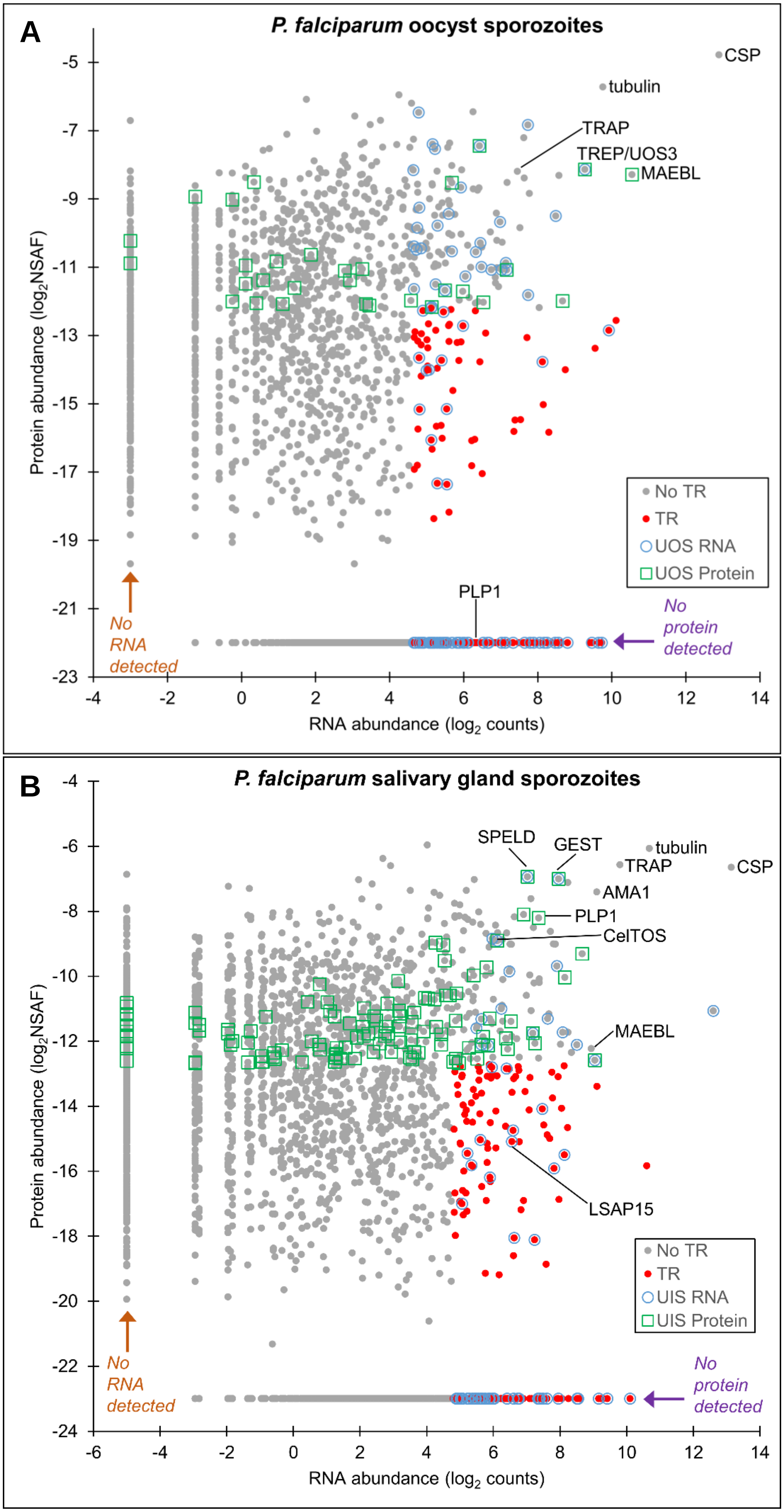

A
P. falciparum oocyst sporozoites
CSP
tubulin
TRAP
TREP/UOS3
MAEBL
PLP1
No TR
TR
UOS RNA
UOS Protein
No RNA detected
No protein detected
Protein abundance (log2NSAF)
RNA abundance (log2 counts)
-5
-7
-9
-11
-13
-15
-17
-19
-21
-23
-4
-2
0
2
4
6
8
10
12
14
B
P. falciparum salivary gland sporozoites
SPELD
GEST
tubulin
TRAP
CSP
AMA1
PLP1
CelTOS
MAEBL
LSAP15
No TR
TR
UIS RNA
UIS Protein
No RNA detected
No protein detected
Protein abundance (log2NSAF)
RNA abundance (log2 counts)
-4
-6
-8
-10
-12
-14
-16
-18
-20
-22
-24
-6
-4
-2
0
2
4
6
8
10
12
14

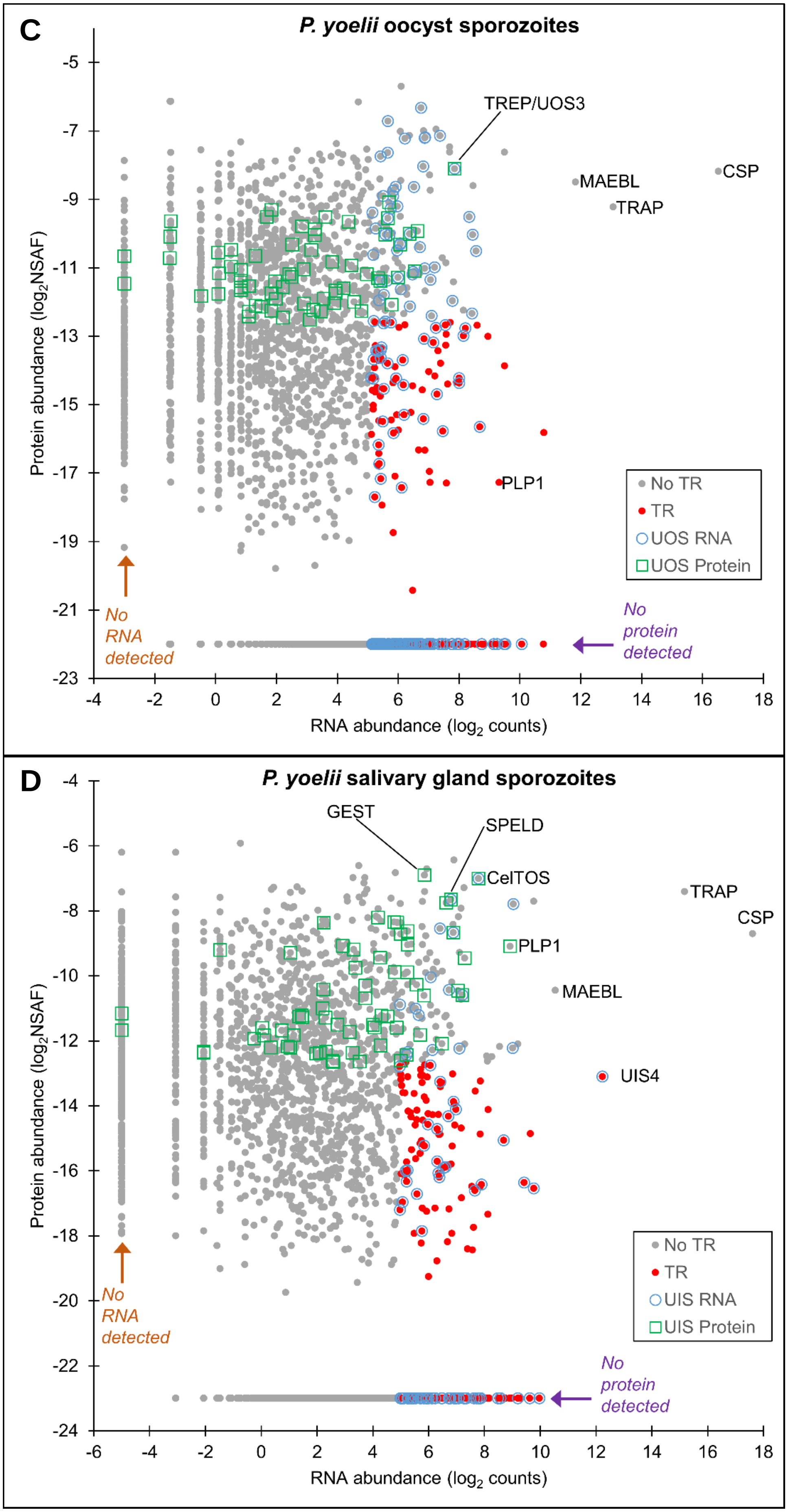
C
P. yoelii oocyst sporozoites
Protein abundance (log2NSAF)
RNA abundance (log2 counts)
TREP/UOS3
MAEBL
CSP
TRAP
PLP1
No TR
TR
UOS RNA
UOS Protein
No RNA detected
No protein detected
D
P. yoelii salivary gland sporozoites
Protein abundance (log2NSAF)
RNA abundance (log2 counts)
GEST
SPELD
CelTOS
TRAP
CSP
PLP1
MAEBL
UIS4
No TR
TR
UIS RNA
UIS Protein
No RNA detected
No protein detected

Supplementary Figure 2: Comparison of transcript and protein levels in oocyst sporozoites and salivary gland sporozoites. Transcript abundance is presented as log2 of the average reads and protein abundance is presented as the $\log_2$ of the normalized spectral abundance factor (NSAF). All genes detected as either mRNA or protein in each sample type are plotted (closed gray circles) for A) *P. falciparum* oocyst sporozoites, B) *P. falciparum* salivary gland sporozoites, C) *P. yoelli* oocyst sporozoites, and D) *P. yoelii* salivary gland sporozoites. Transcripts that we define as translationally repressed (TR), i.e., transcript is in top decile of abundance but protein is in lower 50th percentile of abundance or undetected, are shown as closed red circles. Additionally, transcripts that we define as upregulated in infectious sporozoites (UIS) or upregulated in oocyst sporozoites (UOS) are marked with open blue circles in the salivary gland sporozoite and oocyst sporozoite plots, respectively, and UIS and UOS proteins are marked with open green squares in the salivary gland sporozoite and oocyst sporozoite plots, respectively. Notable proteins are labeled. Transcript and protein levels are seen to correlate well for a core set of conserved, high-abundance, essential proteins (e.g., CSP, TRAP), whereas the subset of transcripts we identify as translationally repressed in salivary gland sporozoites include proteins with known liver stage function (see Supplementary Figure 1).

Supplementary Table 1: The top 25 most abundant mRNAs in oocyst sporozoites and salivary gland sporozoites of *Plasmodium falciparum and Plasmodium yoelii*.

***Plasmodium* Gene ID**	**Gene Name or Symbol**	**Product Description**	**oo-spz Rank[a]**	**sg-spz Rank[a]**	**UIS or UOS[b]**
PF3D7_0304600	CSP	Circumsporozoite protein	1	1	
PF3D7_1147800	MAEBL	MAEBL, membrane associated erythrocyte binding-like protein	2	14	
PF3D7_1239600	ThzK	hydroxyethylthiazole kinase	3	21	
PF3D7_0404800		conserved Plasmodium protein, unknown function	4	94	UOS
PF3D7_1008700		tubulin beta chain	5	3	
PF3D7_0829800		unspecified product	6	123	UOS
PF3D7_0315700		conserved Plasmodium membrane protein, unknown function	7	74	UOS
PF3D7_1361100	SEC24A	Sec24A, protein transport protein	8	32	
PF3D7_0809100	SpzPfEMP1	SpzPfEMP1	9	104	UOS
PF3D7_1026600		conserved Plasmodium protein, unknown function	10	4	
PF3D7_1442600	TREP	TREP/UOS3, TRAP-like protein	11	252	UOS
PF3D7_0918200		50S ribosomal protein L3, apicoplast, putative	12	1846	UOS
PF3D7_0404900	P41	p41, 6-cysteine protein	13	45	
PF3D7_0919400	PDI9	protein disulfide isomerase 9	14	9	
PF3D7_0515300	PI3K	PI3K, phosphatidylinositol 3-kinase	15	84	
PF3D7_1015900	ENO	enolase	16	44	
PF3D7_1030100	PRP22	PRP22, pre-mRNA-splicing factor ATP-dependent RNA helicase, putative	17	1119	UOS
PF3D7_0820600		conserved Plasmodium membrane protein, unknown function	18	22	
PF3D7_1338300	EF-1g	elongation factor 1-gamma, putative	19	1254	UOS
PF3D7_0721200		conserved Plasmodium protein, unknown function	20	270	UOS
PF3D7_1033100	AdoMetDC /ODC	S-adenosylmethionine decarboxylase/ornithine decarboxylase	21	8	
PF3D7_0925700	HDAC1	HDAC1, histone deacetylase 1	22	25	
PF3D7_0709000	CRT	CRT, chloroquine resistance transporter	23	1290	UOS
PF3D7_0525700		conserved Plasmodium protein, unknown function	24	279	UOS

PF3D7_1023900	CHD1	CHD1, chromodomain-helicase-DNA-binding protein 1 homolog, putative	25	11	
PF3D7_0304600	CSP	CSP, circumsporozoite protein	1	1	
PF3D7_1235200	VP2	V-type K+-independent H+-translocating inorganic pyrophosphatase	322	2	UIS
PF3D7_1008700		tubulin beta chain	5	3	
PF3D7_1026600		conserved Plasmodium protein, unknown function	10	4	
PF3D7_1475800		conserved Plasmodium protein, unknown function	37	5	UIS
PF3D7_1335900	TRAP	TRAP, thrombospondin-related anonymous protein	47	6	
PF3D7_0516500	MFS1	major facilitator superfamily domain-containing protein, putative	788	7	UIS
PF3D7_1033100	AdoMetDC /ODC	S-adenosylmethionine decarboxylase/ornithine decarboxylase	21	8	
PF3D7_0919400	PDI9	protein disulfide isomerase 9	14	9	
PF3D7_1347200	NT1	nucleoside transporter 1	529	10	UIS
PF3D7_1023900	CHD1	CHD1, chromodomain-helicase-DNA-binding protein 1 homolog, putative	25	11	
PF3D7_1133400	AMA1	AMA1, apical membrane antigen 1	34	12	
PF3D7_0616500	TLP	TLP, TRAP-like protein	204	13	UIS
PF3D7_1147800	MAEBL	MAEBL, membrane associated erythrocyte binding-like protein	2	14	
PF3D7_0511400		conserved Plasmodium protein, unknown function	80	15	
PF3D7_1365600		DNA topoisomerase VI, b subunit, putative	61	16	
PF3D7_0529200		sugar transporter, putative	371	17	UIS
PF3D7_0407500		mitochondrial carrier protein, putative	118	18	UIS
PF3D7_0102800		conserved Plasmodium protein, unknown function	202	19	UIS
PF3D7_1020800	DLAT	dihydrolipoamide acyltransferase component E2	116	20	UIS
PF3D7_1239600	ThzK	hydroxyethylthiazole kinase	3	21	
PF3D7_0820600		conserved Plasmodium membrane protein, unknown function	18	22	
PF3D7_1346100	SEC61	SEC61, protein transport protein, subunit alpha	87	23	
PF3D7_1407700		conserved Plasmodium protein, unknown function	40	24	
PF3D7_0925700	HDAC1	HDAC1, histone deacetylase 1	22	25	

PY17X_0405400	CSP	circumsporozoite (CS) protein	1	1	
PY17X_1354800	TRAP	thrombospondin-related anonymous protein	2	2	
PY17X_0902700	MAEBL	membrane associated erythrocyte binding-like protein	3	4	
PY17X_0624100		RNA-binding protein, putative	4	8	
PY17X_0303200		3'-5' exonuclease, putative	5	18	
PY17X_1024600		conserved Plasmodium protein, unknown function	6	178	UOS
PY17X_0822500		50S ribosomal protein L3, apicoplast, putative	7	1377	UOS
PY17X_0826700	PGK	phosphoglycerate kinase, putative	8	6	
PY17X_0902800		ATP synthase subunit delta, mitochondrial, putative	9	28	
PY17X_0207300		lipid/sterol:H+ symporter, putative	10	119	UOS
PY17X_1007700	PLP1	sporozoite micronemal protein essential for cell traversal	11	17	
PY17X_0314800		conserved Plasmodium protein, unknown function	12	135	UOS
PY17X_0307300		transporter, putative	13	229	UOS
PY17X_1239500		ribosomal large subunit pseudouridylate synthase, putative	14	67	
PY17X_0823600		protein disulfide isomerase, putative	15	33	
PY17X_0826600		para-aminobenzoic acid synthetase, putative	16	22	
PY17X_0712000		conserved Plasmodium protein, unknown function	17	31	
PY17X_0208200		conserved Plasmodium protein, unknown function	18	3887	UOS
PY17X_0405000		EH domain-containing protein, putative	19	230	UOS
PY17X_0611200		conserved Plasmodium protein, unknown function	20	128	
PY17X_0710400		conserved Plasmodium protein, unknown function	21	130	
PY17X_0839000	PKAc	cAMP-dependent protein kinase catalytic subunit	22	496	UOS
PY17X_0611400		general transcription factor 3C polypeptide 5, putative	23	122	
PY17X_0307200		conserved Plasmodium protein, unknown function	24	136	
PY17X_0835500		conserved Plasmodium protein, unknown function	25	3886	UOS
PY17X_0405400	CSP	circumsporozoite (CS) protein	1	1	
PY17X_1354800	TRAP	thrombospondin-related anonymous protein	2	2	

PY17X_0502200	UIS4	early transcribed membrane protein	2489	3	UIS
PY17X_0902700	MAEBL	membrane associated erythrocyte binding-like protein	3	4	
PY17X_0504800		conserved Plasmodium protein, unknown function	1125	5	UIS
PY17X_0826700	PGK	phosphoglycerate kinase, putative	8	6	
PY17X_1218100		conserved Plasmodium protein, unknown function	224	7	UIS
PY17X_0624100		RNA-binding protein, putative	4	8	
PY17X_1117800		AP-2 complex subunit alpha, putative	108	9	UIS
PY17X_1338000		conserved Plasmodium protein, unknown function	466	10	UIS
PY17X_1125300	SMS1	sphingomyelin synthase 1, putative	43	11	
PY17X_1008900	ALP5b	actin-like protein, putative	196	12	UIS
PY17X_1336100		conserved Plasmodium protein, unknown function	51	13	
PY17X_0712100	HSP70	heat shock protein 70	204	14	UIS
PY17X_0837000	CSE1	importin alpha re-exporter, putative	286	15	UIS
PY17X_1119000		trafficking protein particle complex subunit 6A, putative	47	16	
PY17X_1007700	PLP1	sporozoite micronemal protein essential for cell traversal	11	17	
PY17X_0303200		3'-5' exonuclease, putative	5	18	
PY17X_0302700		conserved Plasmodium protein, unknown function	48	19	
PY17X_0511900		conserved Plasmodium protein, unknown function	46	20	
PY17X_1354300		conserved Plasmodium protein, unknown function	3866	21	UIS
PY17X_0826600		para-aminobenzoic acid synthetase, putative	16	22	
PY17X_0505100		tRNA methyltransferase, putative	1408	23	UIS
PY17X_0943700	RTCB	tRNA-splicing ligase RtcB, putative	67	24	
PY17X_0101400	UIS7	conserved rodent malaria protein, unknown function	1133	25	UIS

[a]Abundance rank (1 is highest) for mRNAs measured in oocyst sporozoites (oo-spz) and salivary gland sporozoites (sg-spz).

[b]Transcripts are defined as Upregulated in Oocyst Sporozoites (UOS) or Upregulated in Infectious Sporozoites (UIS) if their abundance is in the top decile and their fold change is >5-fold in one sporozoite stage compared to the other. A complete listing of mRNA abundances and fold-changes between sporozoite stages is provided in Supplementary Data 1.

Supplementary Table 2: The top 25 most abundant proteins in oocyst sporozoites and salivary gland sporozoites of *Plasmodium falciparum* and *Plasmodium yoelii.*

***P. falciparum* Gene ID**	**Gene Name or Symbol**	**Product Description**	**oo-spz Rank[a]**	**sg-spz Rank[a]**	**UIS or UOS[b]**
PF3D7_0304600	CSP	circumsporozoite (CS) protein	1	6	
PF3D7_1008700	N/A	tubulin beta chain	2	2	
PF3D7_1105000	H4	histone H4	3	1	
PF3D7_1105100	H2B	histone H2B	4	5	
PF3D7_1365900	N/A	ubiquitin-60S ribosomal protein L40	5	20	
PF3D7_1246200	ACT1	actin I	6	11	
PF3D7_0816500	HSP20	small heat shock protein HSP20, putative	7	8	
PF3D7_1462800	GAPDH	glyceraldehyde-3-phosphate dehydrogenase	8	12	
PF3D7_1222300	GRP94	endoplasmin, putative	9	33	
PF3D7_1357000, PF3D7_1357100	N/A	elongation factor 1-alpha;elongation factor 1-alpha	10	7	
PF3D7_0903700	N/A	alpha tubulin 1	11	15	
PF3D7_0818200	14-3-3I	14-3-3 protein	12	14	
PF3D7_1350600	N/A	conserved Plasmodium protein, unknown function	13	68	
PF3D7_0819600	N/A	conserved Plasmodium protein, unknown function	14	44	
PF3D7_0408600	SIAP1	sporozoite invasion-associated protein 1	15	22	
PF3D7_0818900	HSP70	heat shock protein 70	16	34	
PF3D7_0917900	HSP70-2	heat shock protein 70	17	46	
PF3D7_1342600	MyoA	myosin A	18	17	
PF3D7_1406800	GAPM3	glideosome associated protein with multiple membrane spans 3	19	43	
PF3D7_1323700	GAPM1	glideosome associated protein with multiple membrane spans 1	20	29	
PF3D7_0503400	ADF1	actin-depolymerizing factor 1	21	3	
PF3D7_0931300	N/A	conserved Plasmodium protein, unknown function	22	243	UOS
PF3D7_0423500	GAPM2	glideosome associated protein with multiple membrane spans 2	23	51	
PF3D7_0919300	TrxL1	thioredoxin-like protein 1, putative	24	18	
PF3D7_1222700	GAP45	glideosome-associated protein 45	25	49	
PF3D7_1105000	H4	histone H4	3	1	

PF3D7_1008700	N/A	tubulin beta chain	2	2	
PF3D7_0503400	ADF1	actin-depolymerizing factor 1	21	3	
PF3D7_1335900	TRAP	thrombospondin-related anonymous protein	49	4	
PF3D7_1105100	H2B	histone H2B	4	5	
PF3D7_0304600	CSP	circumsporozoite (CS) protein	1	6	
PF3D7_1357000, PF3D7_1357100	N/A	elongation factor 1-alpha;elongation factor 1-alpha	10	7	
PF3D7_0816500	HSP20	small heat shock protein HSP20, putative	7	8	
PF3D7_1137800	SPELD	sporozoite surface protein essential for liver stage development	632	9	UIS
PF3D7_1449000	GEST	gamete egress and sporozoite traversal protein, putative	N.D.	10	UIS
PF3D7_1246200	ACT1	actin I	6	11	
PF3D7_1462800	GAPDH	glyceraldehyde-3-phosphate dehydrogenase	8	12	
PF3D7_1407700	N/A	conserved Plasmodium protein, unknown function	63	13	
PF3D7_0818200	14-3-3I	14-3-3 protein	12	14	
PF3D7_0903700	N/A	alpha tubulin 1	11	15	
PF3D7_1438900	Trx-Px1	thioredoxin peroxidase 1	28	16	
PF3D7_1342600	MyoA	myosin A	18	17	
PF3D7_0919300	TrxL1	thioredoxin-like protein 1, putative	24	18	
PF3D7_1133400	AMA1	apical membrane antigen 1	108	19	
PF3D7_1365900	N/A	ubiquitin-60S ribosomal protein L40	5	20	
PF3D7_1324900	LDH	L-lactate dehydrogenase	31	21	
PF3D7_0408600	SIAP1	sporozoite invasion-associated protein 1	15	22	
PF3D7_1017500	ELC	essential light chain ELC	57	23	
PF3D7_0814600	N/A	conserved Plasmodium protein, unknown function	43	24	
PF3D7_1444800	FBPA	fructose-bisphosphate aldolase	96	25	
PY17X_1210100	N/A	tubulin beta chain, putative	1	5	
PY17X_1134800, PY17X_1134900	EF-1alpha	elongation factor 1-alpha, putative	2	2	
PY17X_0944400	N/A	histone H4, putative	3	4	
PY17X_0714500	HSP20	small heat shock protein HSP20, putative	4	11	
PY17X_1330200	GAPDH	glyceraldehyde-3-phosphate dehydrogenase	5	1	
PY17X_1411000	N/A	RNA-binding protein, putative	6	34	
PY17X_1361400	MyoA	myosin A	7	31	
PY17X_0712100	HSP70	heat shock protein 70, putative	8	35	
PY17X_1461900	ACT1	actin I, putative	9	6	

PY17X_0404800	IMC1a	inner membrane complex protein 1a, putative	10	46	
PY17X_0420500	N/A	alpha tubulin 1	11	80	
PY17X_0712800	14-3-3I	14-3-3 protein, putative	12	13	
PY17X_0823500	TrxL1	thioredoxin-like protein 1, putative	13	9	
PY17X_0822200	HSP70-2	heat shock protein 70, putative	14	44	
PY17X_0944300	H2B	histone H2B, putative	15	15	
PY17X_1424900	N/A	conserved Plasmodium protein, unknown function	16	3	
PY17X_1369100	N/A	conserved Plasmodium protein, unknown function	17	140	
PY17X_1306600	TPx1	thioredoxin peroxidase, putative	18	21	
PY17X_0718900	N/A	small GTP-binding protein sar1, putative	19	187	
PY17X_1007600	SIAP1	sporozoite invasion-associated protein 1	20	40	
PY17X_0826700	PGK	phosphoglycerate kinase, putative	21	30	
PY17X_1207600	ALBA3	DNA/RNA-binding protein Alba 3, putative	22	43	
PY17X_0808800	HSP90	heat shock protein 90, putative	23	93	
PY17X_1205100	IMC1c	inner membrane complex protein 1c, putative	24	20	
PY17X_1114100	PNP	purine nucleoside phosphorylase, putative	25	26	
PY17X_1330200	GAPDH	glyceraldehyde-3-phosphate dehydrogenase	5	1	
PY17X_1134800, PY17X_1134900	EF-1alpha	elongation factor 1-alpha, putative	2	2	
PY17X_1424900	N/A	conserved Plasmodium protein, unknown function	16	3	
PY17X_0944400	N/A	histone H4, putative	3	4	
PY17X_1210100	N/A	tubulin beta chain, putative	1	5	
PY17X_1461900	ACT1	actin I, putative	9	6	
PY17X_1316500	GEST	gamete egress and sporozoite traversal protein, putative	N.D.	7	UIS
PY17X_1312400	ALDO2	fructose-bisphosphate aldolase 2	69	8	
PY17X_0823500	TrxL1	thioredoxin-like protein 1, putative	13	9	
PY17X_1434600	CelTOS	cell traversal protein for ookinetes and sporozoites	1461	10	UIS
PY17X_0714500	HSP20	small heat shock protein HSP20, putative	4	11	
PY17X_1344800	LDH	L-lactate dehydrogenase, putative	40	12	
PY17X_0712800	14-3-3I	14-3-3 protein, putative	12	13	
PY17X_1036900	N/A	conserved Plasmodium protein, unknown function	101	14	
PY17X_0944300	H2B	histone H2B, putative	15	15	
PY17X_1143100	N/A	ubiquitin-60S ribosomal protein L40, putative	30	16	
PY17X_1220800	H2A.Z	histone H2A.Z, putative	26	17	

PY17X_1354800	TRAP	thrombospondin-related anonymous protein	154	18	
PY17X_1213100	N/A	conserved Plasmodium protein, unknown function	155	19	
PY17X_1205100	IMC1c	inner membrane complex protein 1c, putative	24	20	
PY17X_1306600	TPx1	thioredoxin peroxidase, putative	18	21	
PY17X_0713100	HMGB2	high mobility group protein B2	66	22	
PY17X_0930100	PGM1	phosphoglycerate mutase, putative	49	23	
PY17X_1422300	H2B.Z	histone H2B variant, putative	74	24	
PY17X_0810800	RPS11	40S ribosomal protein S11, putative	35	25	

[a]Abundance rank (1 is highest) for proteins measured in oocyst sporozoites (oo-spz) and salivary gland sporozoites (sg-spz). N.D., not detected.

[b]Proteins are defined as Upregulated in Oocyst Sporozoites (UOS) or Upregulated in Infectious Sporozoites (UIS) if their abundance is in the top half and their fold change is >5-fold in one sporozoite stage compared to the other. A complete listing of protein abundances using label-free quantification and fold-changes between sporozoite stages is provided in Supplementary Data 1.

Supplementary Table 3: Selected gene products under the translational repression in oocyst sporozoite to UIS protein regulatory program (TR-oospz to UIS protein program) in *Plasmodium yoelii* and/or *Plasmodium falciparum*[a].

***P. falciparum* Gene ID**	***P. yoelii* Gene ID**	**Gene Name or Symbol**	**Product Description**	**Species[b]**	**Gene Deletion Phenotypes[c]**	**Refs[c]**
PF3D7_0408700	PY17X_1007700	PLP1/SPECT2	perforin-like protein 1	*Pf & Py*	Host Traversal Defect	[37], [65]
PF3D7_1218000	PY17X_1436000	TRAMP	thrombospondin-related apical membrane protein	*Pf & Py*		
PF3D7_0511400	PY17X_1112100	N/A	conserved Plasmodium protein	*Pf & Py*		
PF3D7_0616500	PY17X_1117200	TLP	TRAP-like protein	*Pf*	Host Traversal Defect	[52]
PF3D7_1116700	PY17X_0933300	DPAP1	dipeptidyl aminopeptidase 1	*Pf*		
PF3D7_1328800	PY17X_1348600	SIR2A	transcriptional regulatory protein sir2a	*Pf*		
PF3D7_1430100	PY17X_1016100	PTPA	serine/threonine protein phosphatase 2A activator	*Pf*		
PF3D7_0108300	PY17X_0206600	ARP	conserved Plasmodium protein	*Pf*		
PF3D7_0925200	PY17X_0829300	RRP8	ribosomal RNA-processing protein 8	*Pf*		
PF3D7_1110400	PY17X_0939200	N/A	RNA-binding protein	*Pf*		
PF3D7_1147700	PY17X_0902800	N/A	ATP synthase subunit delta, mitochondrial	*Pf*		
PF3D7_0405700	PY17X_1004800	N/A	lysine decarboxylase	*Pf*		
PF3D7_0723300	PY17X_0623500	N/A	conserved Plasmodium protein	*Pf*		

PF3D7_0814700	PY17X_1424800	N/A	conserved Plasmodium protein	*Pf*		
PF3D7_1023800	PY17X_0509100	N/A	conserved Plasmodium protein	*Pf*		
PF3D7_1207400	PY17X_0608400	N/A	conserved Plasmodium protein	*Pf*		
PF3D7_1407600	PY17X_1037000	N/A	conserved Plasmodium protein	*Pf*		
PF3D7_1216600	PY17X_1434600	CelTOS	cell traversal protein for ookinetes and sporozoites	*Py*	Host Traversal Defect	[53], [63], [64]
PF3D7_0805200	PY17X_1228600	GAMER	gamete release protein	*Py*	Reduced Infectivity of Host via Mosquito Bite	[67]
PF3D7_1030200	PY17X_0515300	CLAMP	claudin-like apicomplexan microneme protein	*Py*		
PF3D7_0818400	PY17X_0712600	FCF1	rRNA-processing protein FCF1	*Py*		
PF3D7_1342100	PY17X_1360900	IRP	aconitate hydratase	*Py*		
PF3D7_1024100	PY17X_0509400	N/A	conserved Plasmodium protein	*Py*		
PF3D7_1434400	PY17X_1012000	N/A	conserved Plasmodium membrane protein	*Py*		
PF3D7_0624800	PY17X_1125100	N/A	conserved Plasmodium protein	*Py*		
PF3D7_1213400	PY17X_1431400	N/A	conserved Plasmodium protein	*Py*		
PF3D7_1235100	PY17X_1452200	N/A	conserved Plasmodium protein	*Py*		

[a]Select gene products designated as translationally repressed (TR) in oocyst sporozoites for which the protein is also observed to be upregulated in salivary gland sporozoites (UIS). A complete list of proteins meeting these criteria is provided in Supplementary Data 6.

[b]The gene product was found to be in the TR-oospz to UIS protein program in either *P. falciparum* only (Pf), *P. yoelii* only (*Py*), or both species (*Pf & Py*).

[c]Previously observed sporozoite phenotypes.

Supplementary Table 4: Selected gene products under the Pan-Sporozoite translational repression program in *Plasmodium yoelii* and *Plasmodium falciparum*[a].

P. falciparum Gene ID	*P. yoelii* Gene ID	Gene Name or Symbol	Product Description	*P. falciparum*[b]	*P. yoelii*[b]
PF3D7_1007700	PY17X_1209100	ApiAP2-I	AP2 domain transcription factor AP2-I	OO+SG	SG-only
PF3D7_1023900	PY17X_0509200	CHD1	chromodomain-helicase-DNA-binding protein 1	OO+SG	OO+SG
PF3D7_0801900	PY17X_1231800	LSD2	lysine-specific histone demethylase	OO+SG	OO+SG
PF3D7_1031200	PY17X_0516200	MORN1	MORN repeat-containing protein 1	OO+SG	SG-only
PF3D7_1107300	PY17X_0942100	PAIP1	polyadenylate-binding protein-interacting protein 1	OO+SG	SG-only
PF3D7_1146000	PY17X_0904400	RSA4	ribosome assembly protein 4	OO+SG	SG-only
PF3D7_1008100	PY17X_1209500	UIS11	zinc finger protein	OO+SG	SG-only
PF3D7_0317300	PY17X_0811100	N/A	conserved Plasmodium protein	OO+SG	OO+SG
PF3D7_0207100	PY17X_0305100	N/A	conserved Plasmodium protein, unknown function	OO+SG	SG-only
PF3D7_0404800	PY17X_1003900	N/A	conserved Plasmodium protein, unknown function	OO+SG	OO+SG
PF3D7_1248700	PY17X_1464200	N/A	conserved Plasmodium protein, unknown function	OO+SG	SG-only
PF3D7_0404900	PY17X_1004000	P41	6-cysteine protein P41	OO+SG	
PF3D7_1107800	PY17X_0941600	ApiAP2	AP2 domain transcription factor	OO+SG	
PF3D7_1350900	PY17X_1369400	ApiAP2-O4	AP2 domain transcription factor AP2-O4	OO+SG	
PF3D7_1456000	PY17X_1323500	ApiAP2	AP2 domain transcription factor, unnamed	OO+SG	
PF3D7_1323000	PY17X_1342900	FabZ	beta-hydroxyacyl-ACP dehydratase	OO+SG	

PF3D7_0217500	PY17X_0314700	CDPK1	calcium-dependent protein kinase 1	OO+SG	
PF3D7_1122800	PY17X_0927500	CDPK6	calcium-dependent protein kinase 6	OO+SG	
PF3D7_1103800	PY17X_0945600	NOT1	CCR4-NOT transcription complex subunit 1	OO+SG	
PF3D7_1235300	PY17X_1452400	NOT4	CCR4-NOT transcription complex subunit 4	OO+SG	
PF3D7_0806300	PY17X_1227600	FLP	ferlin-like protein	OO+SG	
PF3D7_0805200	PY17X_1228600	GAMER	gamete release protein	OO+SG	
PF3D7_1116800	PY17X_0933200	HSP101	heat shock protein 101	OO+SG	
PF3D7_0925700	PY17X_0829800	HDAC1	histone deacetylase 1	OO+SG	
PF3D7_1308900	PY17X_1409100	DCP2	mRNA-decapping enzyme 2	OO+SG	
PF3D7_1106000	PY17X_0943400	RUVB2	RuvB-like helicase 2	OO+SG	
PF3D7_0420300	PY17X_0523100	ApiAP2	AP2 domain transcription factor		SG-only
PF3D7_0730300	PY17X_0215800	AP2-L	AP2 domain transcription factor AP2-L		OO+SG
PF3D7_0622900	PY17X_1123200	AP2-SP3	AP2 domain transcription factor AP2-SP3		SG-only
PF3D7_1241800	PY17X_1457700	DBP9	ATP-dependent RNA helicase DBP9		SG-only
PF3D7_0821300	PY17X_0709700	DHX36	ATP-dependent RNA helicase DHX36		OO+SG
PF3D7_0905700	PY17X_0418500	ATG3	autophagy-related protein 3		SG-only
PF3D7_0415300	PY17X_0717500	CRK3	cdc2-related protein kinase 3		OO+SG
PF3D7_1241500	PY17X_1457400	UIS8	conserved Plasmodium protein, unknown function		SG-only
PF3D7_1016900	PY17X_0502200	UIS4 (Only *P. yoelii*)	early transcribed membrane protein		SG-only
PF3D7_1312900	PY17X_1413100	EIF4G	eukaryotic translation initation factor 4 gamma		SG-only
PF3D7_0810800	PY17X_1428800	PPPK-DHPS	hydroxymethyldihydro pterin pyrophosphokinase-		SG-only

			dihydropteroate synthase		
PF3D7_1427100	PY17X_1019000	UIS28	lipase, putative		SG-only
PF3D7_1028700	PY17X_0513900	MTRAP	merozoite TRAP-like protein		OO+SG
PF3D7_1332500	PY17X_1352300	N/A	SAM-dependent RNA methyltransferase		SG-only
PF3D7_0616500	PY17X_1117200	TLP	TRAP-like protein		SG-only

[a]Select gene products that are designated as translationally repressed (TR) in both oocyst sporozoites and salivary gland sporozoites in either or both *P. falciparum* and *P. yoelii*. A complete list of proteins meeting these criteria is provided in Supplementary Data 6.

[b]OO+SG, gene product was TR in both oocyst sporozoites (OO) and salivary gland sporozoites (SG). SG only, gene product was designated as TR in salivary gland sporozoites but not oocyst sporozoites. Blank entries indicate gene product was not TR in that species.
