# Supplementary material for: Transcriptomics and proteomics reveal two waves of translational repression during the maturation of malaria parasite sporozoites
Source: Nat Commun. 2019 Oct 31;10:4964. doi: 10.1038/s41467-019-12936-6 (PMC6823429; doi:10.1038/s41467-019-12936-6)
Supplement: Supplementary file 4 — Description of Additional Supplementary Files [file 41467_2019_12936_MOESM4_ESM.docx]

**Supplementary Data 1:** Complete transcriptomic and proteomic datasets and their analyses.

**Supplementary Data 2:** UOS and UIS mRNAs from *P. falciparum* and *P. yoelii*.

**Supplementary Data 3:** Transcripts in the top seventh to ninth deciles that increase in abundance 10-fold or greater in salivary gland sporozoites as compared to oocyst sporozoites.

**Supplementary Data 4:** UOS and UIS proteins from *P. falciparum* and *P. yoelii*.

**Supplementary Data 5:** Tryptic peptide analyses of putatively translationally repressed transcripts using the CONSeQuence algorithm.

**Supplementary Data 6:** Extended data and GO terms on translationally repressed transcripts.

**Supplementary Data 7:** Oligonucleotides used in this study.
