# Supplementary material for: Addendum: Transcriptomics and proteomics reveal two waves of translational repression during the maturation of malaria parasite sporozoites
Source: Nat Commun. 2022 Jan 6;13:283. doi: 10.1038/s41467-021-27767-7 (PMC8738726; doi:10.1038/s41467-021-27767-7)
Supplement: Supplementary file 10 — Supplementary Figures S1-S4 [file 41467_2021_27767_MOESM10_ESM.pdf]

**Supplementary Figure 1: Lindner and Swearingen *et al.***

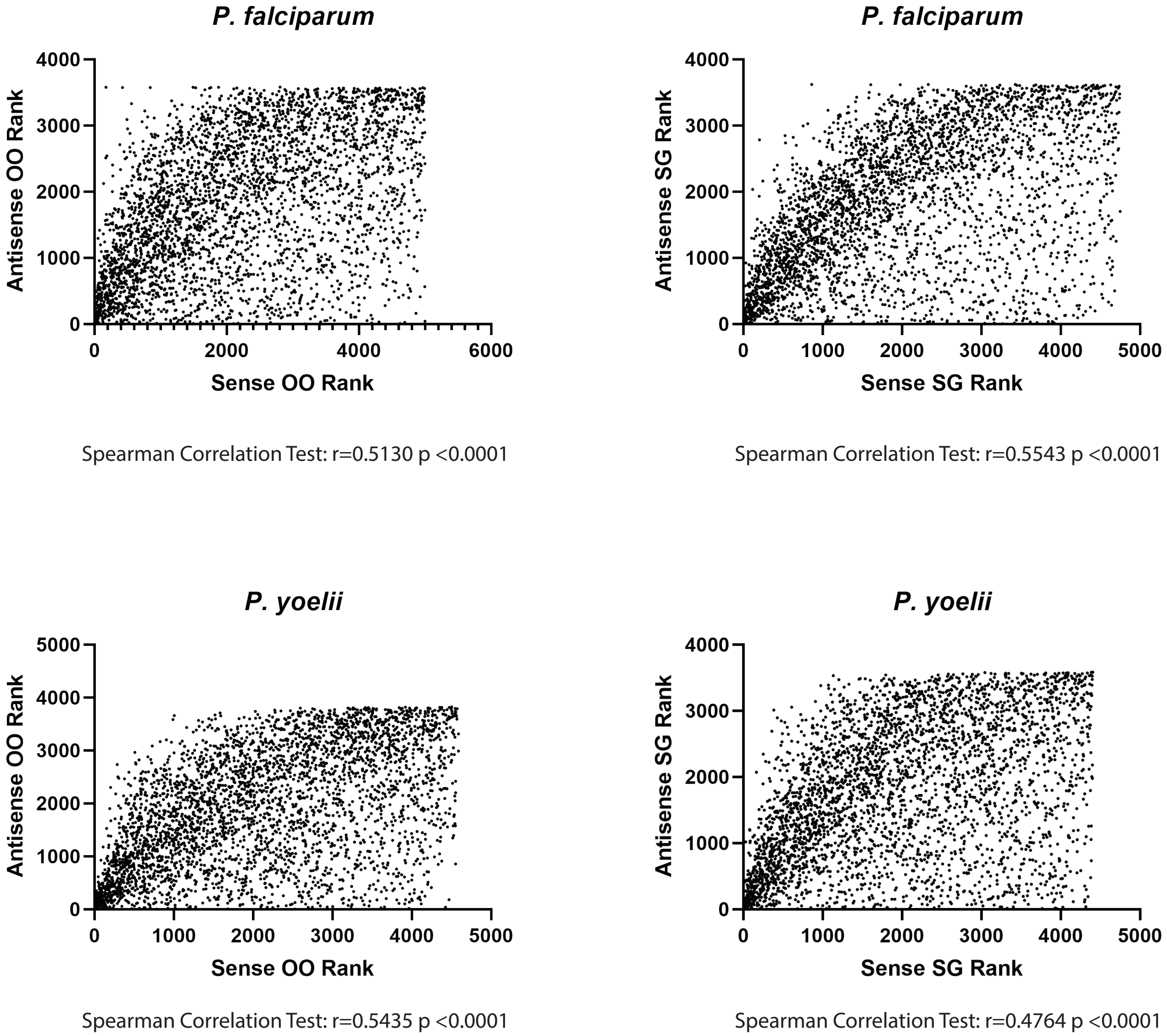


**Supplemental Figure 1: A comparison of the rank abundance of sense and antisense transcripts.** The rank abundances of sense and antisense transcripts from P. falciparum (top panels) and P. yoelii (bottom panels) were plotted for both oocyst sporozoite (OO, left panels) and salivary gland sporozoite (SG, right panels) stages. The correlation between these ranked abundances was assessed by a Spearman correlation test.

**Supplementary Figure 2: Lindner and Swearingen *et al.***

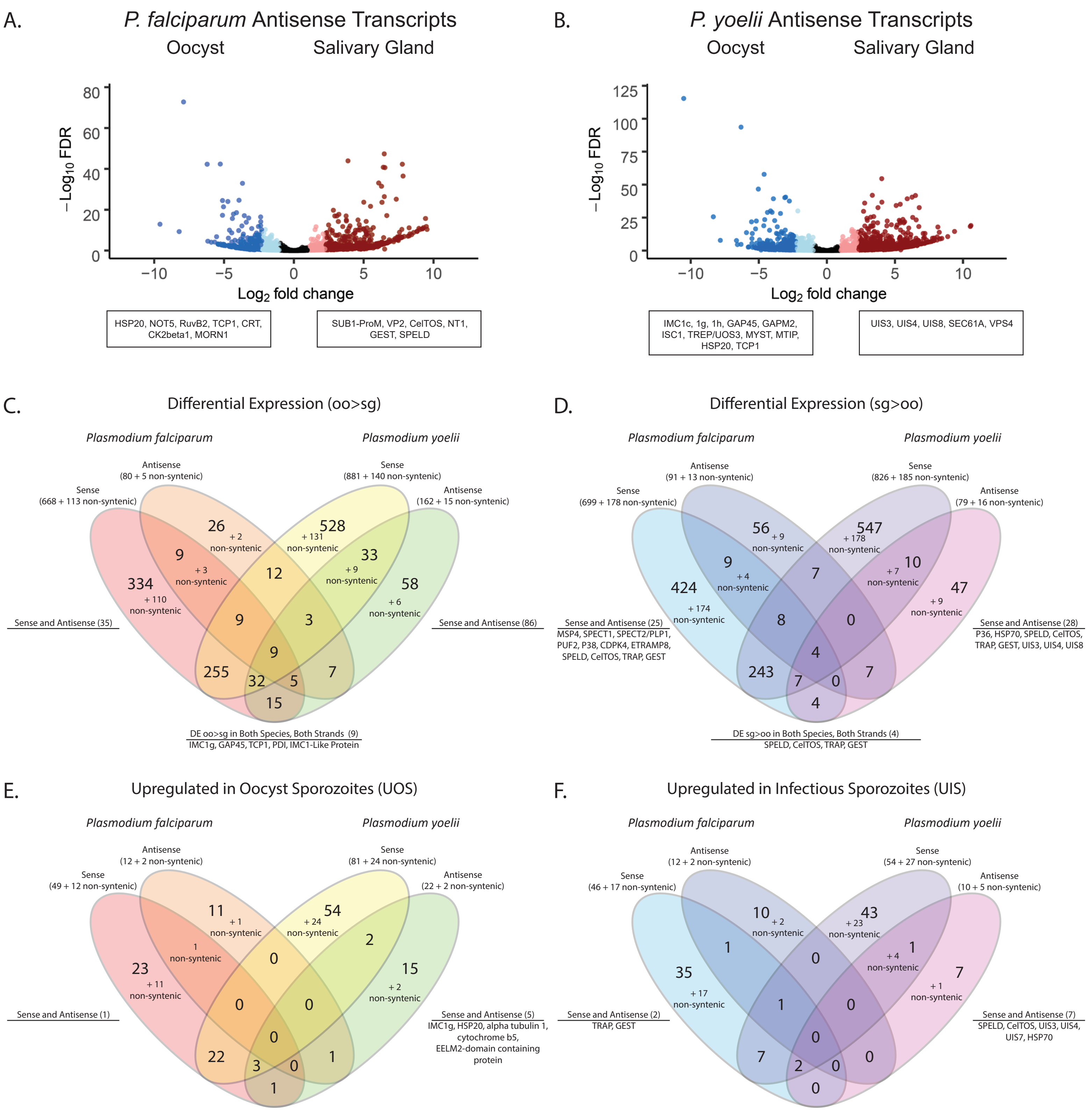


**Supplementary Figure 2: Comparisons of Differentially Expressed, UOS, and UIS Antisense Transcripts.** Volcano plots of antisense transcripts for (A) P. falciparum and (B) P. yoelii illustrate the extent of differential expression across stages of sporozoite development. Venn diagrams of (C) differentially expressed transcripts more abundant in oocyst sporozoites than salivary gland sporozoites (oo>sg), (D) differentially expressed transcripts more abundant in salivary gland sporozoites than oocyst sporozoites (sg>oo), (E) UOS transcripts, and (F) UIS transcripts are illustrated. Transcripts-of-note that are similarly regulated across species, transcriptional levels, and/or sense and antisense strands are indicated.

## Supplementary Figure 3: Lindner and Swearingen *et al.*

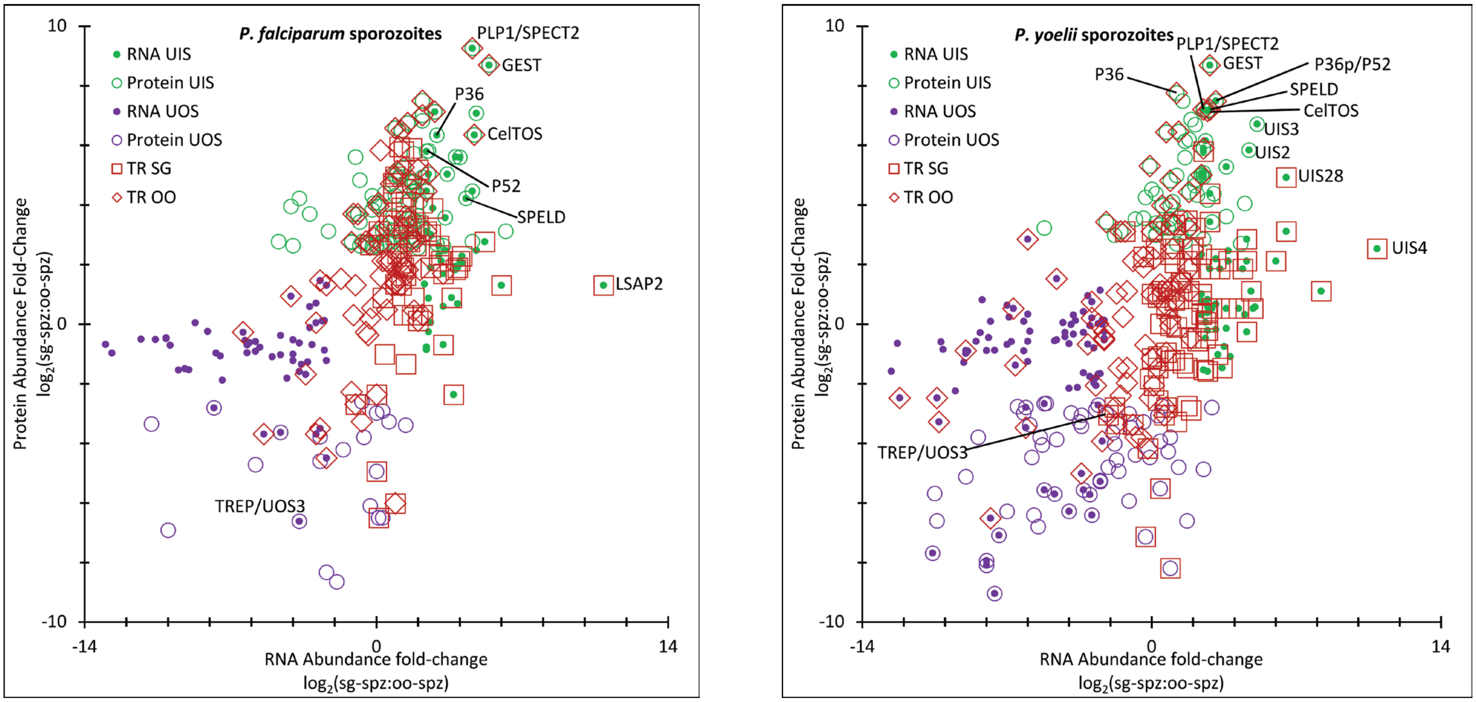


**Supplemental Figure 3. Relative change in protein abundance with respect to relative change in mRNA abundance.** Shown are abundance values for genes that were categorized as at least one of the following: mRNA was upregulated in infectious sporozoites (UIS; filled green circles), protein was UIS (open green circles), mRNA was upregulated in oocyst sporozoites (UOS; closed purple circles), protein was UOS (open purple circles), transcript was translationally repressed in oocyst sporozoites (open red diamonds), or transcript was translationally repressed in salivary gland sporozoites (open red squares). Additionally, data was only plotted if fold-change ratios were obtained for both mRNA and protein. Multiple translational regulation programs can be observed. For example, the invasion-related protein SPELD (sporozoite protein essential for liver stage development) is UIS for both protein and mRNA in both *P. yoelii* and *P. falciparum*. Similarly, the TRAP-like protein TREP (aka UOS3), which is essential for invasion of salivary glands, is UOS by both mRNA and protein in both species. Several other conserved, invasion-related proteins were translationally repressed in oocyst sporozoites, but de-repressed in salivary gland sporozoites, thereby becoming a UIS protein under Program 1 (TR-oospz to UIS Protein) we describe here. Included among these were PLP1/SPECT2 (perforin-like protein 1/sporozoite micronemal protein essential for cell traversal), GEST (gamete egress and sporozoite traversal protein), CelTOS (Cell traversal for ookinetes and sporozoites), and the invasion-related proteins P36 and P52. (Note that P36 and P52 were not designated as translationally repressed in *P. falciparum* because the transcripts were not among the top decile of abundance, but both genes still exhibited abundant transcript with absence of detectable proteins in oocyst sporozoites (Supplemental Table 1)). Proteins under Program 2 (Pan-Sporozoite translational repression program) include LSAP2 (liver stage-associated protein) in *P. falciparum* and UIS4 in *P. yoelii*. Both proteins are important for development of liver stages, and both proteins are mRNAs that are UIS but remain translationally repressed in salivary gland sporozoites, presumably in preparation for de-repression upon arrival in the liver.

**Supplementary Figure 4: Lindner and Swearingen *et al.***

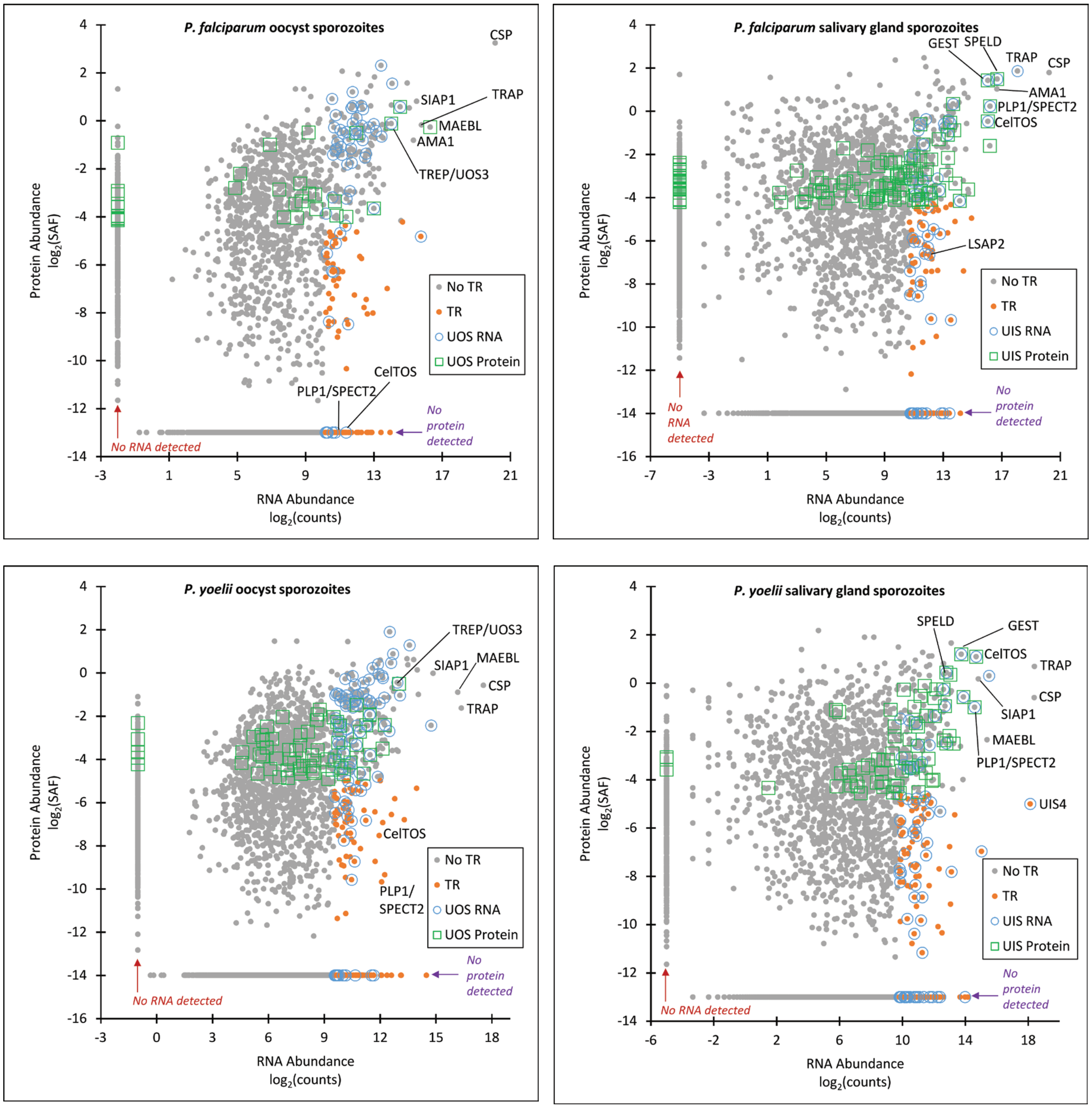


**Supplemental Figure 4: Comparison of transcript and protein abundances in oocyst sporozoites and salivary gland sporozoites.** Transcript abundance is presented as log2 of the average counts (DEseq2) and protein abundance is presented as the log2 of the spectral abundance factor (SAF). All genes detected as either mRNA or protein in each sample type are plotted (closed gray circles) for P. falciparum oocyst sporozoites (top left), P. falciparum salivary gland sporozoites (top right), P. yoelii oocyst sporozoites (bottom left), and P. yoelii salivary gland sporozoites (bottom right). Transcripts that we define as translationally repressed (TR), i.e., transcript is in top decile of abundance but protein is in lower 50th percentile of abundance or is undetected, are shown as closed orange circles. Additionally, transcripts that we define as upregulated in infectious sporozoites (UIS) or upregulated in oocyst sporozoites (UOS) are marked with open blue circles in the salivary gland sporozoite and oocyst sporozoite plots, respectively, and UIS and UOS proteins are marked with open green squares in the salivary gland sporozoite and oocyst sporozoite plots, respectively. Notable proteins are labeled. Transcript and protein levels are seen to correlate well for a core set of conserved, high-abundance, essential proteins, including CSP, TRAP, CelTOS, PLP1/SPECT2, GEST, SPELD, sporozoite invasion-associated protein 1 (SIAP1), and membrane associated erythrocyte binding-like protein (MAEBL). Comparing plots for oocyst sporozoites and salivary gland sporozoites, de-repression of transcripts under Program 1 (TR-oospz to UIS Protein) can be observed for essential invasion-related proteins such as PLP1/SPECT2 and CelTOS. Furthermore, the subset of transcripts we identify as translationally repressed in salivary gland sporozoites include proteins with known liver stage function (e.g. UIS4 and LSAP2), evidence of Program 2 (Pan-Sporozoite Translational Repression program) we describe here.
